# Integrative bioinformatic analysis identifies an extracellular matrix gene signature linked to muscle adaptation to endurance and resistance training

**DOI:** 10.14814/phy2.70874

**Published:** 2026-04-16

**Authors:** Muhammad Isman Sandira, Firman Hasan, Tsubasa Shibaguchi, Hanafi Idris, Mukti Mukhtar, Kazumi Masuda

**Affiliations:** ^1^ Faculty of Human Sciences, Kanazawa University Kanazawa Ishikawa Japan; ^2^ Faculty of Medicine, Hasanuddin University Makassar South Sulawesi Indonesia; ^3^ Institute of Systems, Molecular & Integrative Biology, University of Liverpool Liverpool UK; ^4^ Institute of Liberal Arts and Science, Kanazawa University Kanazawa Ishikawa Japan; ^5^ Institute of Translational Medicine and new Drug Development, China Medical University Taichung City Taiwan; ^6^ Division of Nano Life Science, Graduate School of Frontier Science Initiative, Kanazawa University Kanazawa Ishikawa Japan

**Keywords:** endurance training, exercise gene expression, extracellular matrix remodeling, muscle transcriptome, resistance training, skeletal muscle adaptation

## Abstract

Skeletal muscle exhibits a remarkable adaptive capacity; endurance training enhances mitochondrial capacity, whereas resistance training improves mechanical strength. Despite these differences, both training impose repeated contractile and remodeling demands on skeletal muscle, suggesting a conserved transcriptional response that may underpin the muscle's capacity to endure long‐term training. However, a systematic analysis of the universal gene signature common to both training types has not yet been conducted. We therefore reanalyzed microarray datasets from human skeletal muscle samples obtained before and after endurance and resistance training. We first identified differentially expressed genes in each dataset, then performed a cross‐dataset comparison to determine reproducible transcriptional responses across heterogeneous training protocols. Our results identified extracellular matrix (ECM) remodeling‐related genes as conserved transcriptional responses to both endurance and resistance training. Moreover, we observed modality‐associated gene signatures: endurance training was associated with ECM‐adhesion and matrix connectivity, whereas resistance training preferentially promoted ECM structural maturation and reinforcement. These findings suggest that ECM‐centered transcriptional regulation is a conserved and reproducible feature of skeletal muscle responses to both endurance and resistance training. The central role of ECM remodeling in the plasticity of human skeletal muscle establishes a comprehensive framework for future mechanistic investigations into how exercise induces cellular adaptations.

## 1 INTRODUCTION

Skeletal muscle exhibits remarkable phenotypic plasticity in response to repeated exercise, yet the nature of this adaptation varies depending on the type of training (Hoppeler et al., 2011; McGlory et al., 2017). Endurance exercise is typically characterized by increases in mitochondrial content, oxidative enzyme capacity, and capillary density, reflecting improved metabolic efficiency and fatigue resistance (Befroy et al., 2008; Koma et al., 2024). In contrast, resistance training promotes elevations in myofibrillar protein synthesis, fiber cross‐sectional area, and force output, supporting greater mechanical strength (Damas et al., 2016; McGlory et al., 2017; Vann et al., 2020). These contrasting outcomes demonstrate the wide physiological range of skeletal muscle remodeling in response to long‐term exercise stimuli. Furthermore, the different training modalities engage distinct biological pathways to meet their respective functional demands (Pillon et al., 2020).

Despite these modality‐specific physiological outcomes, both endurance and resistance training impose repeated mechanical and metabolic demands on overlapping skeletal muscle tissue (Liu et al., 2010; Viggars et al., 2023). Such recurrent stimuli activate a series of conserved cellular processes related to stress sensing, repair, and structural maintenance, regardless of whether the primary adaptation is metabolic or hypertrophic adaptation (Beiter et al., 2024; Dickinson et al., 2018; Gehlert et al., 2022). This suggests that some components of the transcriptional response may be shared across exercise types. Identifying these common, load‐related programs is essential because they likely represent the foundational mechanisms that enable muscles to tolerate long‐term training, despite variations in exercise type (Dickinson et al., 2018; Pillon et al., 2020). Distinguishing these universal responses from modality‐specific patterns is therefore necessary for clarifying the core biological principles that govern muscle plasticity.

Although numerous transcriptomic studies have examined exercise adaptation in skeletal muscle, most have evaluated a single training modality, a single cohort, or a specific protocol in isolation (Lavin et al., 2021; Lindholm et al., 2016; Murach & Bagley, 2025). As a result, their findings often differ greatly, reflecting variability in study design, participant characteristics, and biopsy timing (Murach & Bagley, 2025; Pillon et al., 2020). Large‐scale meta‐analyses have integrated multiple cohort studies and significantly advanced this field. One meta‐analysis mainly compared acute and long‐term training responses by pooling across all types of exercise, which can explain the temporal trajectory of transcriptomic adaptation (Amar et al., 2021). Another meta‐analysis explicitly distinguishes between endurance and resistance training, as well as acute and chronic, but predominantly focuses on identifying differentially expressed genes at the gene level (Pillon et al., 2020) Therefore, the presence and composition of a genuinely cross‐study transcriptional program that persists despite differences in protocol, sampling conditions, and analytical platforms remain insufficiently defined (Amar et al., 2021; Pillon et al., 2020; Sun et al., 2025).

**FIGURE 1:**
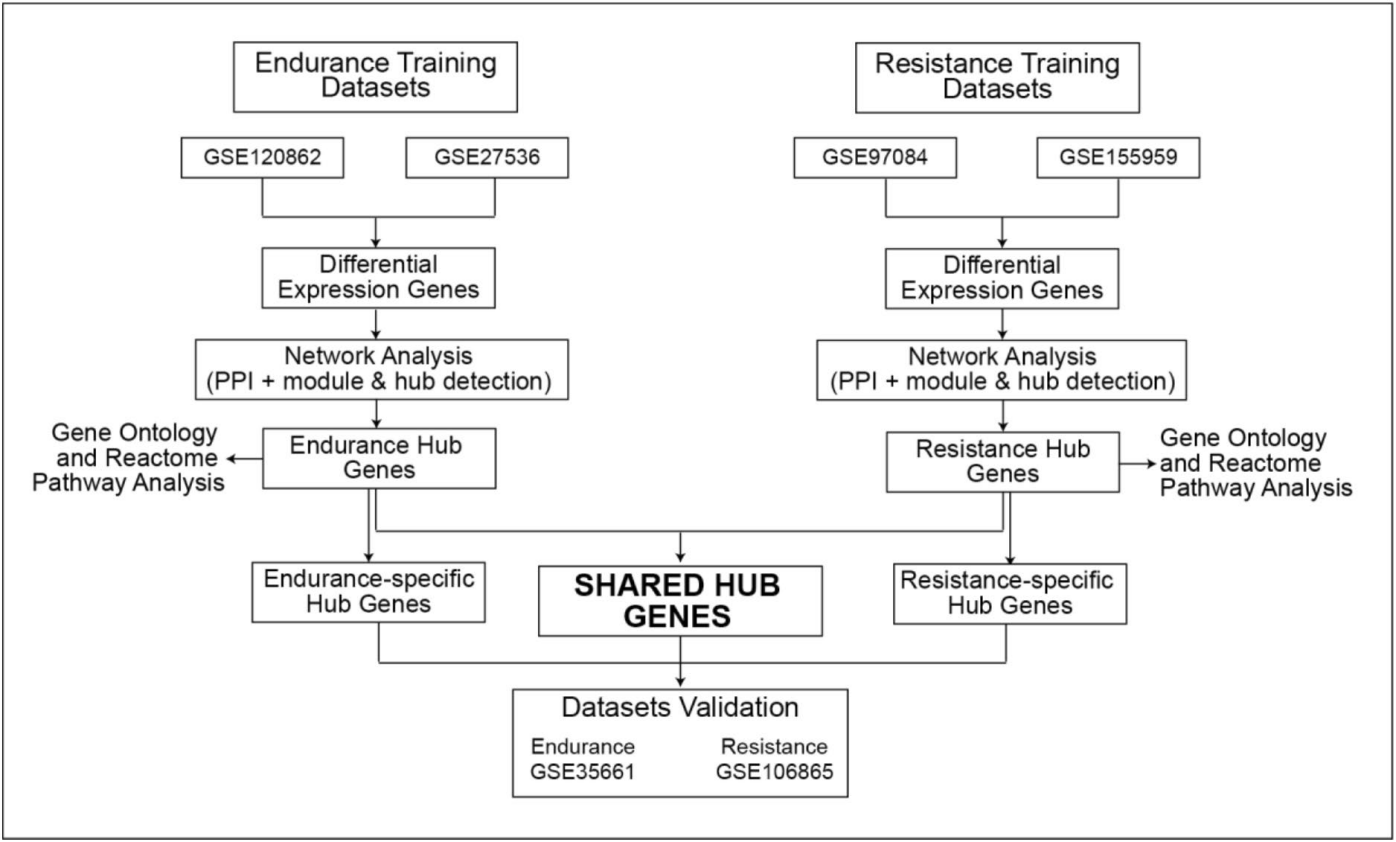
Bioinformatic research workflow in the present study.

To address this gap, we reanalyzed publicly available microarray datasets of human skeletal muscle that included both endurance and resistance training. Using a bioinformatic approach, we combined differential expression analysis with gene‐overlap assessment, functional enrichment, and protein–interaction network construction and compared the resulting patterns with external datasets (Figure 1). This allowed us to identify transcriptional signals that persist across analyzed datasets despite differences in protocol, cohort composition, and analytical approaches. Instead of emphasizing the typical response of each modality, we aimed to identify pathways that are reproducibly regulated under both training conditions.

Using this cross‐dataset framework, we successfully identified a reproducible transcriptional signature across all analyzed cohorts: genes related to extracellular matrix remodeling consistently increased following both long‐term endurance and resistance training. This shared structural signal remained robust despite differences in study design, sampling conditions, and analytical platforms. The central position of these pathways within network‐level analyses distinguished them from the more variable modality‐specific responses. These findings highlight a conserved transcriptional program engaged during long‐term training that provides a foundational understanding of how shared and divergent components of skeletal muscle adaptation are organized.

## 2 MATERIALS AND METHODS

### 2.1 Research design

We performed a cross‐dataset reanalysis of human skeletal muscle microarray studies that compared pre‐ and posttraining conditions for endurance and resistance training. Public data were obtained from the NCBI Gene Expression Omnibus (GEO) (https://www.ncbi.nlm.nih.gov/geo/). The analysis followed a reproducible bioinformatics workflow. Transcriptomic datasets were acquired, and relevant experimental contrasts were defined. Differential expression analysis was conducted using GEO2R based on the *limma* framework (GEO2R/limma) to identify differentially expressed genes. Overlapping gene sets were identified by intersection analysis and visualized using Venn diagrams. Subsequently, functional enrichment was performed using Gene Ontology and Reactome pathway enrichment (GO/Reactome) to characterize associated biological functions and pathways. Protein–protein interaction (PPI) networks were then constructed, followed by module detection and hub gene prioritization to identify key network components. Finally, candidate hub genes were validated using independent datasets and assessed by receiver operating characteristic (ROC) curve analysis. In addition, specific‐modality adaptation genes from previous studies were analyzed and correlated with identified hub genes (Figure 1).

### 2.2 Data acquisition

#### 2.2.1 Endurance training datasets: GSE120862 and GSE27536

In GSE120862, seven untrained males underwent knee extension training on one leg at 65% maximum force, 1 h/day, 5 days/week for 2 months. A biopsy of the vastus lateralis muscle was performed at baseline and 48 h posttraining (Popov et al., 2019). In addition, GSE27536 analyzed the COPD patient and healthy individuals who underwent endurance training (constant force at 70%) for 8 weeks. Our analysis was limited to twelve healthy volunteers by comparing pre‐ and post‐8‐week training (sedentary healthy vs. trained healthy) (Turan et al., 2011).

### 2.3 Resistance training datasets: GSE97084 and GSE155959

In GSE97084, participants in the resistance training group followed a structured program for 12 weeks (4 days/week) that included upper‐body (2 sessions) and lower‐body (2 sessions), targeting the main muscle groups. Each session consisted of 8–12 repetitions of each exercise with progressively increasing load until nearing volitional fatigue. The muscle biopsy was collected from the vastus lateralis 72 h after the last bout. We compared ten young pre‐ and posttraining participants who underwent resistance training only (Robinson et al., 2017). In GSE155959, participants performed leg extension training and unilateral leg press three times a week for 10 weeks. The training program was planned to induce muscle hypertrophy. We only analyzed 12 young pre‐ and posttraining (after 10 weeks) before the unloading phase (Stokes et al., 2020).

### 2.4 Differential expression and intersection (Venn) analysis

Group contrasts were performed for each dataset by using GEO2R (https://www.ncbi.nlm.nih.gov/geo/geo2r/), which implements limma linear modeling (Ritchie et al., 2015). Platform annotation mapped the probe to the official gene symbol. A differentially expressed gene (DEG) was defined as an adjusted *p* <0.05 and |log2FC| >0.5. Overlap of DEGs between datasets was analyzed and visualized using the FunRich application (version 3.1.3) (http://funrich.org/download) (Pathan et al., 2015).

### 2.5 Functional enrichment (gene ontology and Reactome)

The functional annotation was performed using the Database for Annotation, Visualization, and Integrated discovery (DAVID) v2025 (https://davidbioinformatics.nih.gov) that provides a comprehensive set of functional annotation tools to interpret the biological impact of large gene lists. By using DAVID, we analyze the Gene Ontology (GO), namely Biological Process (BP), Cellular Component (CC), and Molecular Function (MF), and the Reactome pathway (Huang et al., 2009a, 2009b). The significance threshold was FDR <0.05 and Count ≥3. All terms/pathways were reported and ordered based on *p*‐value. The top five terms from each GO and the top 10 Reactome pathways were shown in the main figures, with the complete results available in the Data S1 and S2. Enrichment dot/bubble plots were generated using SRplot, an online scientific plotting platform (https://www.bioinformatics.com.cn/en) (Tang et al., 2023).

### 2.6 Protein–protein interactions (PPI), modules, and hub genes

Connectivity among DEGs was analyzed using STRING v12 (https://string‐db.org/), a database that systematically predicts PPIs, both physical interactions and functional associations. We constructed the PPI network with default STRING settings (minimum interaction score: 0.4 and all interaction sources enabled) (Szklarczyk et al., 2023). The PPI network was then visualized and further analyzed using Cytoscape v3.8.2 (Doncheva et al., 2019). The dense subnetwork was identified using the Molecular Complex Detection (MCODE) plugin (Bader & Hogue, 2003) with the following parameters: *K*‐score = 2, node cutoff = 0.2, maximum depth = 100. To prioritize the central genes and integrate network‐module evidence, Cytohubba (Chin et al., 2014) was used, and the PPI‐MCODE module was combined with Cytoscape.

### 2.7 Validation of the external cohort and ROC analysis

External validation for endurance training was performed using GSE35661 (24 participants) (Keller et al., 2011), while GSE106865 (ten participants) (Damas et al., 2018) was used for resistance training validation. RStudio was used for the Wilcoxon and Spearman correlation analyses for both datasets. Discrimination between pre‐ and posttraining was evaluated using ROC analysis in SPSS v27, and the results were reported as AUC values. The violin charts for the Wilcoxon test were designed using GraphPad Prism 8.

### 2.8 Specific‐modality adaptation genes analysis

The specific‐modality adaptation genes were selected from previous references. Using RStudio, we analyzed the expression levels (Wilcoxon) of these genes and their correlations (Spearman correlation) with our hub genes in GSE35661 for endurance and GSE106865 for resistance training. The violin plots were created in GraphPad Prism 8, and the correlation heatmap was created in RStudio (version 4.4.2).

### 2.9 Reproducibility and software

All datasets lacked subject identity and are openly accessible in GEO. All analyses leveraged widely used software, including GEO/GEO2R/limma, DAVID, STRING, Cytoscape/MCODE/CytoHubba, RStudio, and SPSS. The thresholds, parameters, versions, and URLs were cited to ensure reproducibility. All figures were prepared in Adobe Illustrator.

## 3 RESULTS

### 3.1 Identification of differentially expressed genes (DEGs) across datasets

**FIGURE 2:**
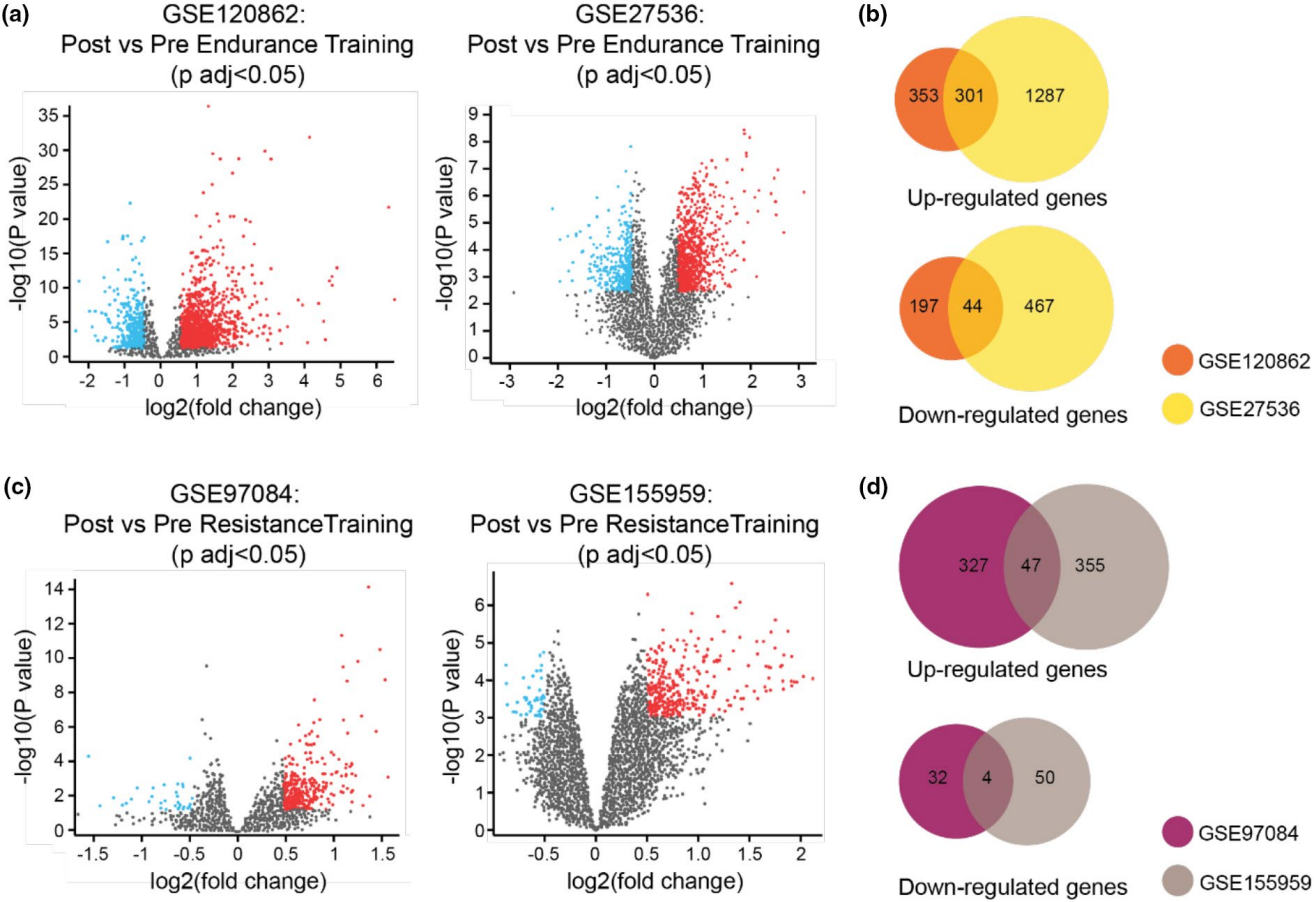
Differential expression gene analysis of endurance and resistance training. (a) Volcano plot analysis of Post versus Pre Endurance training of two datasets using the GEO2R online tool. There are 1112 DEGs (837 increasing and 275 decreasing) in GSE27536 and 2099 DEGs (1588 increasing and 511 decreasing) in GSE120862. (b) The Venn diagram shows the overlapping DEGs of GSE120862 and GSE27536. We found 301 increases and 44 decreases overlapping DEG genes in these datasets. (c) Volcano plot analysis of Post versus Pre Resistance training from two datasets, GSE 97084 and GSE155959. We observe a total of 370 DEGs (337 increasing and 33 decreasing) in GSE97084 and 405 DEGs (355 increasing and 50 decreasing) in GSE155959. (d) The Venn diagram identifies 51 DEGs overlapping genes in datasets, with 47 genes increasing while 4 genes decreasing. All DEGs are defined by an adjusted *p*‐value <0.05 and |log2FC| ≥ 0.5.

Using the GEO2R online tool, we identified 1112 DEGs (837 increasing and 275 decreasing) in GSE27536 and 2099 DEGs (1588 increasing and 511 decreasing) in GSE120862 that were differentially expressed between post‐ and preendurance training, as shown in Figure 2a. Furthermore, 370 DEGs (337 increasing and 33 decreasing) and 405 DEGs (355 increasing and 50 decreasing) were identified in GSE97084 and GSE155959, respectively (Figure 2c). Further analysis of these DEGs using a Venn diagram revealed 345 overlapping DEGs in endurance training, including 301 genes that increased and 44 that decreased, as shown in Figure 2b. On the other hand, 51 DEGs were identified in resistance training, with 47 genes increasing and only four decreasing (Figure 2d). All related genes are listed in Data S1 and S2.

### 3.2 Analysis of functional enrichment of DEGs

**FIGURE 3:**
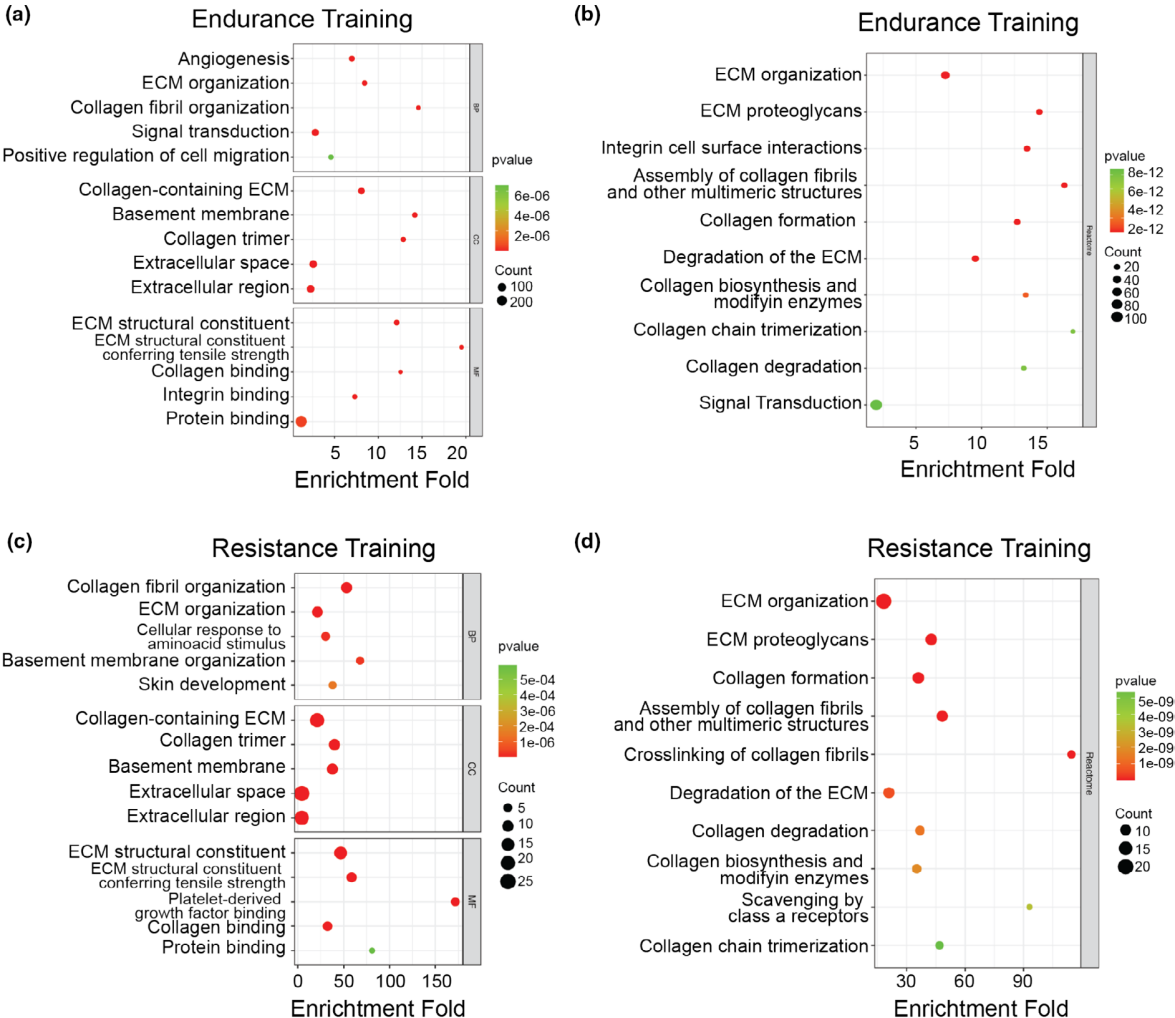
The Gene Ontology (GO) and Reactome enrichment analysis of endurance and resistance DEGs using the David database. (a) The GO and (b) Reactome enrichment analysis of endurance training reveal that the collagen turnover and ECM organization consistently appear. (c) The GO and (d) Reactome pathway in resistance training also show the collagen turnover and ECM organization as prominent pathways. BP, biological process; CC, cellular component; MF, molecular function. The pathways are sorted based on *p*‐value.

To further explain the biological significance of the overlapping DEGs identified in both training modalities, we conducted enrichment analysis of GO and Reactome using the DAVID database. In the endurance analysis, 345 overlapping DEGs were significantly enriched in biological processes (BP), including angiogenesis (1.6 × 10^−16^), extracellular matrix organization (ECM) (4.3 × 10^−14^), collagen fibril organization (1.7 × 10^−13^), signal transduction (4.6 × 10^−13^), and positive regulation of cell migration (1.6 × 10^−8^). For the cellular component category (CC), the enrichment was dominated by collagen‐containing ECM (9.6 × 10^−30^), basement membrane (3.1 × 10^−18^), collagen trimer (1.56 × 10^−16^), extracellular space (1.46 × 10^−14^), and extracellular region (6.8 × 10^−13^). Analysis of molecular function (MF) showed that ECM structural constituent (4.4 × 10^−19^), ECM structural constituent conferring tensile strength (1.36 × 10^−14^), collagen binding (1.08 × 10^−11^), integrin binding (3.44 × 10^−11^), and protein binding (4.02 × 10^−9^) were dominant, as shown in Figure 3a and Data S1. Moreover, Reactome pathway analysis identified that DEGs were mainly associated with ECM organization, Integrin cell surface interactions, collagen formation, and signal transduction pathways (Figure 3b and Data S1).

In resistance training, GO analysis showed that enrichment BP included collagen fibril organization (4.53 × 10^−12^), ECM organization (7.16 × 10^−9^), cellular response to amino acid stimulus (2.01 × 10^−5^), basement membrane organization (2.56 × 10^−5^), and skin development (1.46 × 10^−4^). CC enrichments were dominated by collagen‐containing ECM (1.5 × 10^−20^), Collagen trimer (2.43 × 10^−12^), Basement membrane (7.63 × 10^−11^), Extracellular space (7.72 × 10^−11^), and Extracellular region (2.36 × 10^−8^). The terms of MF were mainly connected with ECM structural constituent (5.29 × 10^−20^), ECM structural constituent conferring tensile strength (1.7 × 10^−9^), Platelet‐derived growth factor binding (1.3 × 10^−8^), Collagen binding (1.03 × 10^−6^), and Protein binding (5.9 × 10^−4^) as shown in Figure 3c and Data S2. In the Reactome analysis, the pathways related to ECM organization and Collagen formation were dominant (Figure 3d and Data S2).

### 3.3 Identified hub gene by protein–protein interaction (PPI) network analysis

**FIGURE 4:**
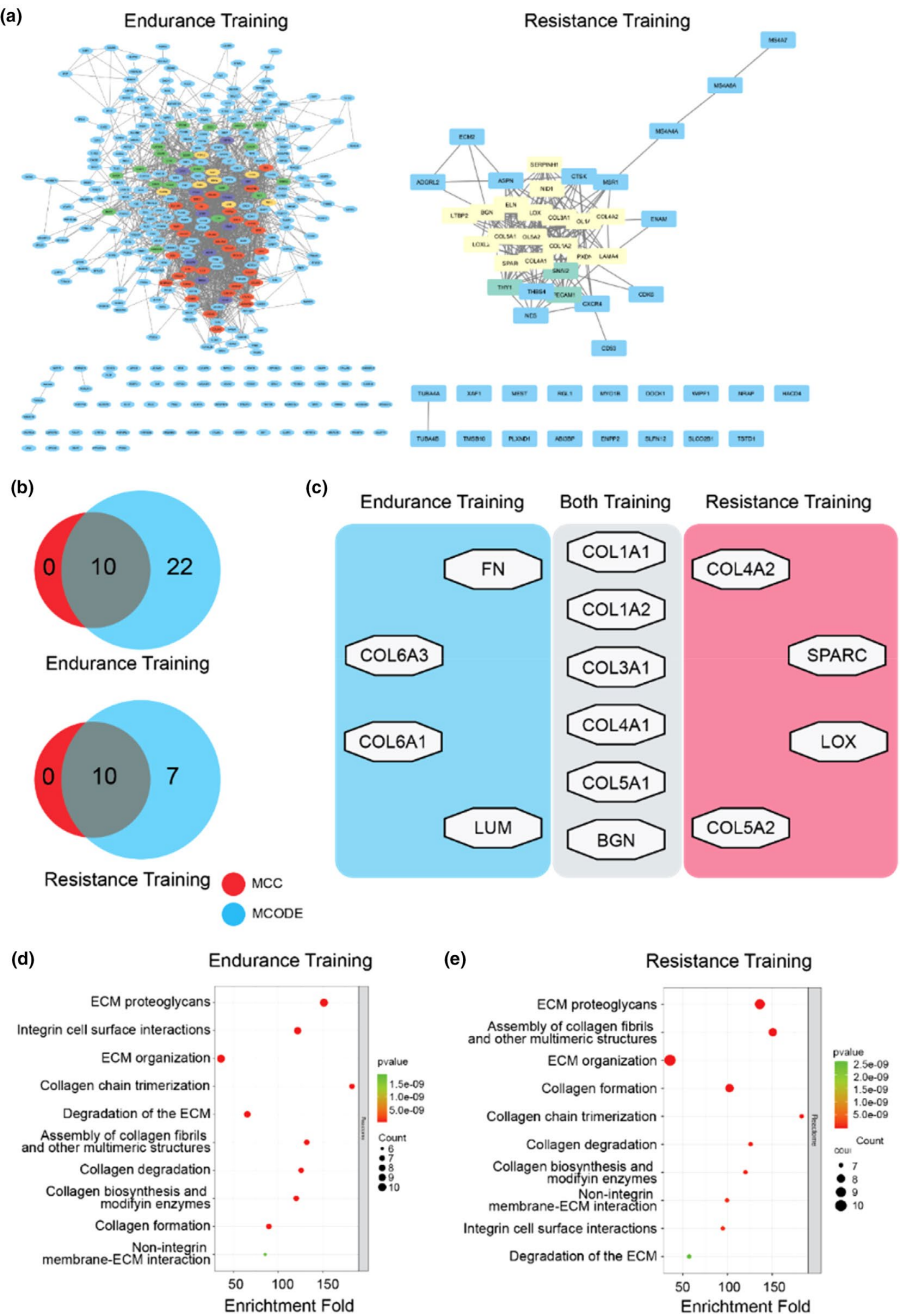
Identification of the gene hubs by Protein–Protein Interactions analysis, as well as the Reactome enrichment of these hub genes. (a) PPI network of Endurance (left) and Resistance (right) Hub Genes that Cytoscape visualizes. Using MCODE tools, we identified four MCODE clusters for endurance training and two MCODE clusters for resistance training. The most significant clusters are 340 nodes and 422 edges for endurance training and 17 nodes and 116 edges for resistance training. (b) The Venn diagram of MCC versus MCODE genes. Ten genes overlap between MCC and MCODE in both training modalities. (c) List of the hub genes of endurance (blue), resistance (red), and both (gray) training. Six genes overlap between these two types of exercise: *COL1A1* (collagen type I alpha 1 chain), *COL1A2* (collagen type I alpha 2 chain), *COL3A1* (collagen type III alpha 1 chain), *COL4A1* (collagen type IV alpha 1 chain), *COL5A1* (collagen type V alpha 1 chain), and *BGN* (biglycan). The Reactome enrichment of (d) Endurance training and (e) Resistance training using the David database. All the top ten pathways are the same, dominated by ECM remodeling and collagen turnover.

To investigate the interactions among identified DEGs, we constructed a PPI network using the STRING database. Using that method, we found 340 nodes and 1871 edges for endurance training and 51 nodes and 204 edges for resistance training. These networks were visualized using Cytoscape software. Module analysis used the MCODE plugin to identify the most significant submodules: 34 nodes and 422 edges for endurance training, and 17 nodes and 116 edges for resistance training (Figure 4a).

To identify the hub genes in these networks, the CytoHubba plugin was applied using topology algorithms, including MCC, to rank the top 10 genes. After placing the top 10 genes in MCC for each training modality, we integrated them with MCODE data using a Venn diagram to identify overlapping genes between MCODE and MCC, which we subsequently termed hub genes (Figure 4b). We identified 10 hub genes, of which six genes overlapped between endurance and resistance training: *COL1A1* (collagen type I alpha 1 chain), *COL1A2* (collagen type I alpha 2 chain), *COL3A1* (collagen type III alpha 1 chain), *COL4A1* (collagen type IV alpha 1 chain), *COL5A1* (collagen type V alpha 1 chain), and *BGN* (biglycan). *COL6A1* (collagen type VI alpha 1 chain), *COL6A3* (collagen type VI alpha 3 chain), *FN1* (Fibronectin 1), and *LUM* (Lumican) were specific to endurance training, whereas *COL4A2* (collagen type IV alpha 2 chain), *COL5A2* (collagen type V alpha 2 chain), *LOX* (Lysyl Oxidase), and *SPARC* (Secreted Protein Acidic and Cysteine‐Rich) were particularly associated with resistance training (Figure 4c).

The subsequent enrichment analysis using the DAVID database showed that these hub genes were significantly connected with the ECM and collagen remodeling pathway. Reactome pathway analysis across both modalities identified pathways that were almost identical, dominated by ECM and collagen organization, including ECM proteoglycans. Integrin cell‐surface interaction was prominent in endurance training, whereas assembly of collagen fibrils and other multimeric structures was dominant in resistance training (Figure 4d,e).

### 3.4 External validation of all hub genes

**FIGURE 5:**
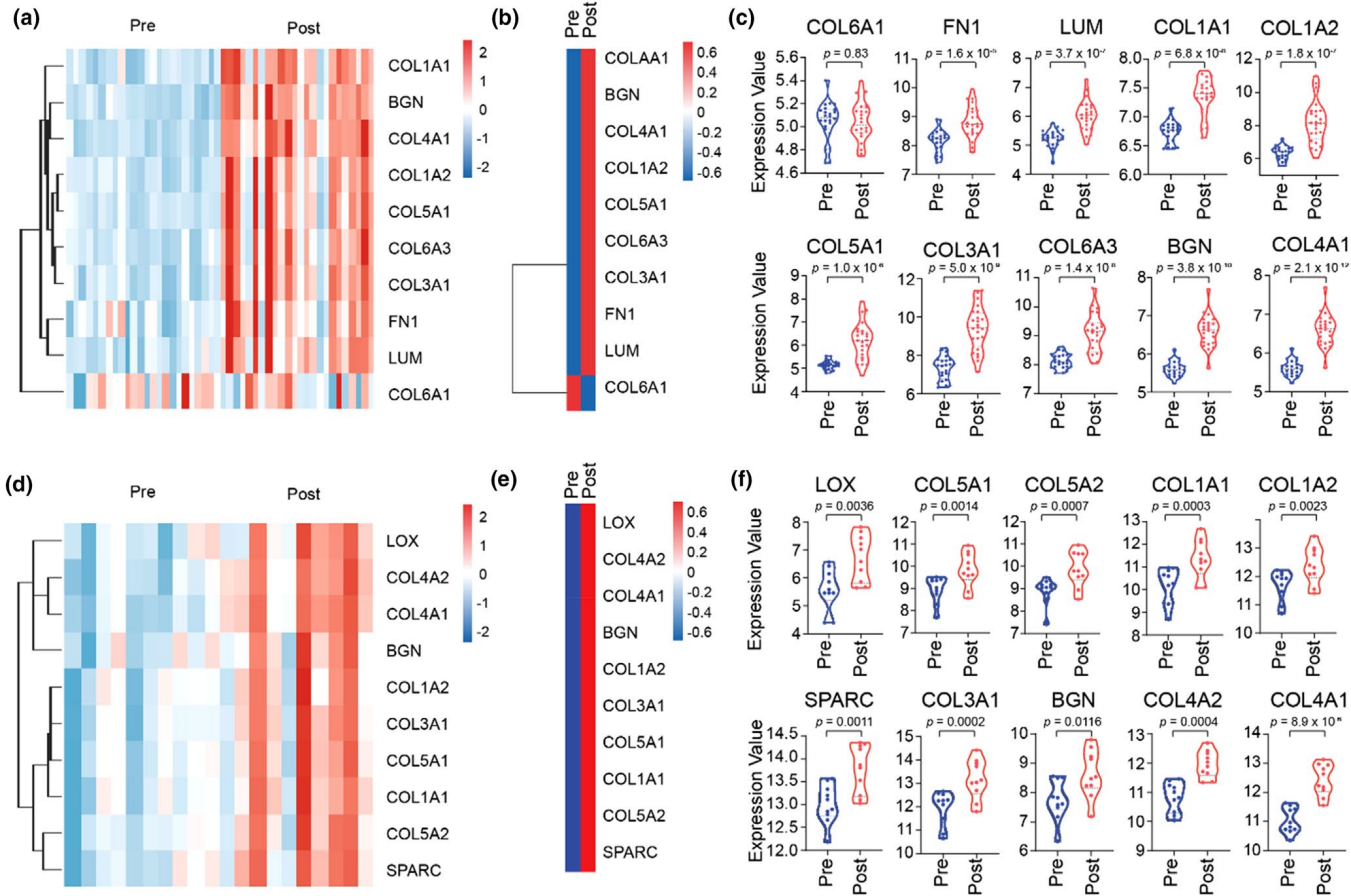
Validation of Hub Genes using another dataset. (a) The gene expression heatmap of 10 hub genes in endurance training from the GSE35661 dataset. We can see a clear color difference between post‐ (red) and pre‐ (blue) training, except in *COL6A1*. (b) The summarized heatmap of all endurance training hub genes. (c) The violin plots of all endurance hub genes. The images show that all genes are significantly increasing, except *COL6A1*. (d) The heatmap of all hub genes' expression in resistance training from the GSE106865 dataset. Despite slight variations, the heatmap shows color trends between post‐ (red) and pre‐ (blue) training. (e) The summarized heatmap of all resistance training hub genes. (f) The violin plots of all resistance hub genes. The bars show that all hub genes are significantly enhanced.

External validation was performed using the other datasets for each training modality: GSE35661 for endurance and GSE106865 for resistance. Wilcoxon analysis was performed in RStudio to assess the expression of the identified hub genes across these datasets. In endurance training, nine out of ten genes significantly increased after exercise, except *COL6A1* (Figure 5a–c). The heatmap showed apparent differences between the pre‐ and posttraining samples. On the other hand, all hub genes increased substantially in resistance training (Figure 5d–f). These data indicated a consistent transcriptional response of our gene hubs in both training modalities.

**FIGURE 6:**
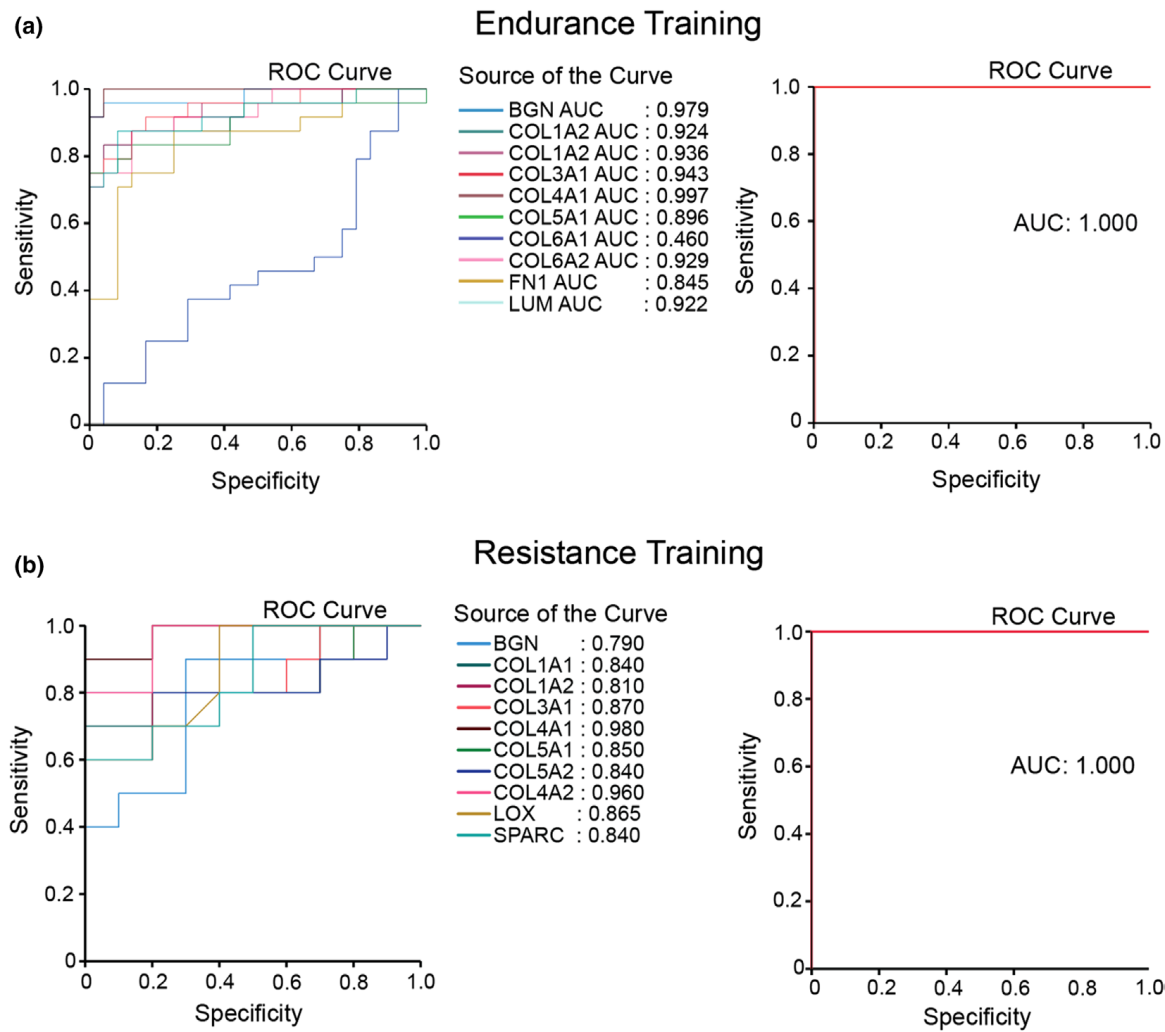
Validation of hub genes using an independent dataset based on Receiver Operating Characteristic (ROC) analysis. (a) ROC curves of individual hub genes in endurance training (Left), showing generally high discriminatory performance between post‐ and pretraining samples from 0.845 to 0.997, except for *COL6A1*, and ROC curve of the collective endurance hub‐gene model (Right), demonstrating perfect separation between post‐ and pretraining conditions (AUC = 1.000), indicating strong combined predictive performance. (b) ROC curves of individual hub genes in resistance training (Left), showing consistently high classification accuracy across genes, although with modest variability in individual AUC values (0.79–0.96), and ROC curve of the collective resistance hub‐gene model (Right), also showing perfect discrimination between post‐ and pretraining samples (AUC = 1.000). AUC, area under the curve; ROC, receiver operating characteristic.

To strengthen our data validation, we applied the Receiver Operating Characteristic (ROC) analysis of our gene hubs using these independent datasets. This method allowed us to assess the discriminatory performance of these genes in distinguishing between pre‐ and postexercise conditions. The gene with a higher area under the curve (AUC) potentially can discriminate well between training conditions. The ROC data indicate that almost all of these genes can effectively differentiate between pre‐ and posttraining conditions. In endurance training, the AUC for each gene ranged from 0.845 to 0.997, except for *COL6A1*, which had an AUC of 0.46 (Figure 6a). Similarly, in the resistance training, the AUC range for each gene was 0.79–0.96 (Figure 6b). Cumulatively, all hub genes across training types could near‐perfectly differentiate between pre‐ and posttraining conditions, with an AUC of 1 (Figure 6a,b).

### 3.5 Modality‐specific ECM remodeling in exercise adaptation

**FIGURE 7:**
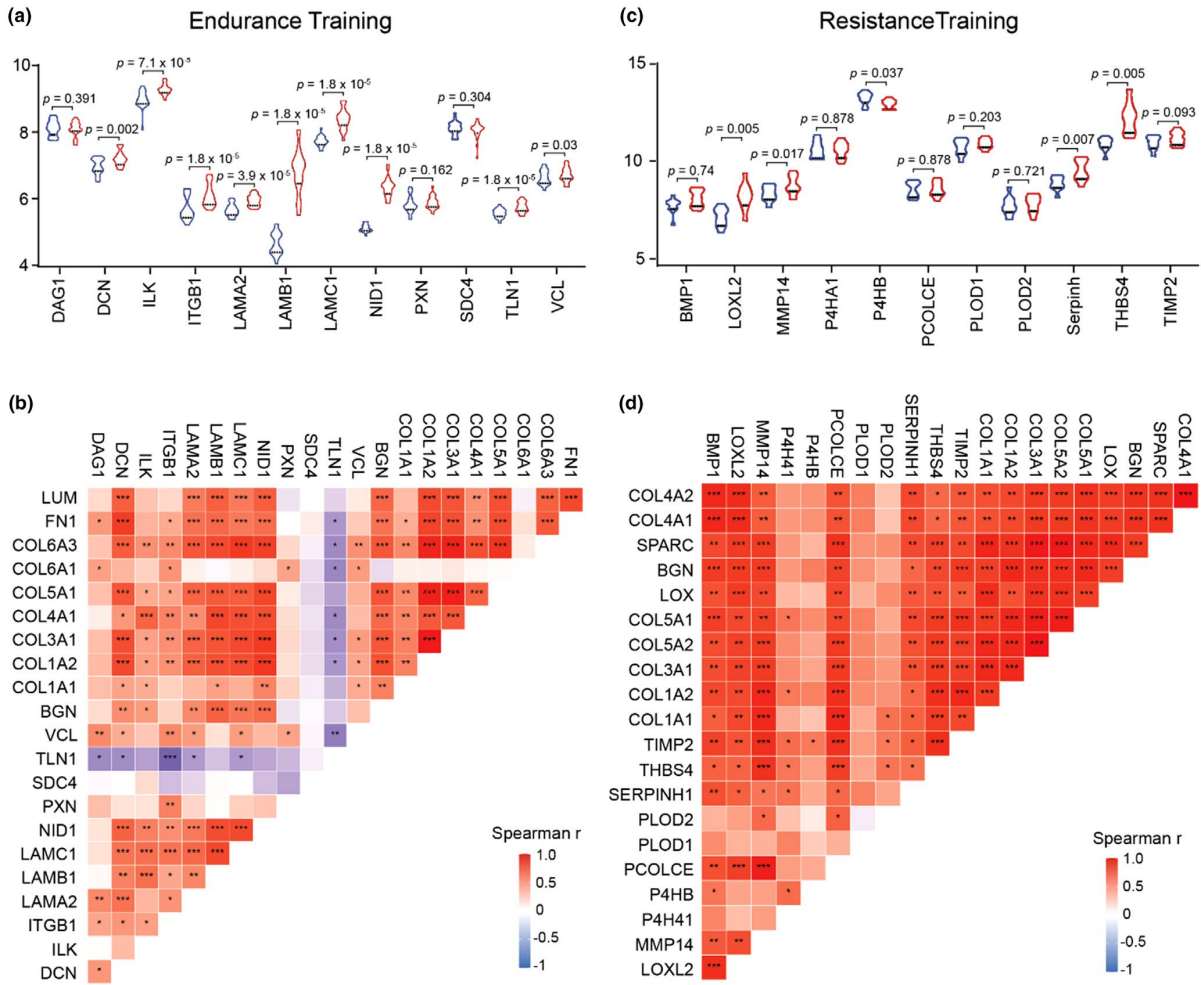
Analysis of genes related to specific modality adaptations in endurance and resistance training. (a) The violin plot of the Wilcoxon analysis of post‐ versus preendurance training. *ITGB1, LAMB1, LAMC1, NID1, TLN1, LAMA2, ILK, DCN*, and *VCL* (*p* <0.05) were significantly increased, while PXN, SDC4, and DAG1 were not (*p* >0.05). (b) Spearman correlation heatmap of postendurance training. Modest correlation (*r* ≈ 0.4–0.7, *p* <0.05) of adhesion mediators (*ITGB1, TLN1, ILK, VCL*), and strong correlation (*r* ≈ 0.6–0.9) of basement membrane components (*LAMA2, LAMB1, LAMC1, NID1*) with our hub genes. (c) The violin plot of the Wilcoxon analysis of post‐ versus preresistance training. *LOXL2, THBS4, MMP14*, and *SERPINH1*, were significantly increased (*p* <0.05), while P4HB tended to decrease (*p* <0.05). The other genes, *BMP1, P4HA1, PLOD1/2*, and *PCOLCE*, did not change (*p* >0.05). (d) Spearman correlation heatmap of postresistance training. A strong correlation (*r* ≈ 0.7–0.95, *p* <0.001) between our hub genes and the cross‐linking enzymes (*LOX, LOXL2*), fibrillogenic regulators (*SERPINH1*), secretion/folding machinery (*THBS4, BMP1*), and remodeling proteases (*MMP14, TIMP2*).

Although several hub genes were shared across both modalities, each training still exhibited typical characteristics that reflected its physiological demands. In endurance training, transcriptional signals were dominated by ECM adhesion, characterized by increased expression of *COL6A1, COL6A3, LUM*, and *FN1*. Previous studies have reported that these genes are crucial for pericellular architecture and integrin‐mediated adhesion, which form an interface for mechanosensing and response in muscle fibers (Cho et al., 2022; Roman et al., 2018; Thorsteinsdóttir et al., 2011; Urciuolo et al., 2013). Consistent with these functions, the enrichment pathways repeatedly positioned collagen assembly, proteoglycan metabolism, and integrin‐ECM interaction among the highest‐order terms in the Reactome. To validate this observation, we identified established components of the pericellular architecture and integrin‐mediated adhesion based on previous references and included the following genes: *ITGB1, ILK, TLN1, VCL, PXN, LAMA2, LAMB1, LAMC1, NID1, SDC4, DAG1, and DCN* (Ahmad et al., 2020; Hashmi & Marinkovich, 2011; Meinen et al., 2007; Rønning et al., 2015; Thorsteinsdóttir et al., 2011). The Wilcoxon analysis of our dataset revealed that *ITGB1, LAMB1, LAMC1, NID1, TLN1, ILK, DCN, VCL*, and *LAMA2* were significantly increased relative to pretraining, while *PXN, SDC4*, and *DAG1* were not changed (Figure 7a).

Furthermore, to identify whether the adhesion‐related genes were coregulated as an integrated structural response, we analyzed Spearman correlations between these genes and our hub genes. The results showed that there was a substantial (r ≈ 0.6–0.9) correlation among the adhesion mediators (*ITGB1, TLN1, ILK, VCL*), basement membrane components (*LAMA2, LAMB1, LAMC1, NID1*), and interstitial effectors such as BGN and collagen chains (*COL1A1, COL1A2, COL3A1, COL4A1, COL5A1, COL5A2, COL6A3*) (Figure 7b). Even though not all adhesion‐related genes showed significant enhancement, the consistency of the association direction supports the idea of a coordinated regulatory program rather than isolated gene expression changes. Biologically, this trend aligns with the strengthening of basement membrane architecture, in which laminin‐nidogen scaffolds interact with integrin β1 via talin and vinculin, increasing mechanical signaling and force transmission during long‐term aerobic loading.

In contrast, resistance training facilitated the transcription program that is associated with collagen cross‐linking and mechanical reinforcement. Four genes that were specific to resistance training, *LOX, SPARC, COL5A2*, and *COL4A2*, reflected the ECM structural maturation, including collagen fibrillogenesis, cross‐linking, and extracellular matrix stabilization, which contribute to tissue mechanical consolidation (Hohenester et al., 2008; Ida et al., 2018; Steffensen et al., 2021; Wenstrup et al., 2004). To evaluate our hypothesis, we investigated the genes associated with that function in the references, including *LOXL2, THBS4, SERPINH1, MMP14, P4HB, BMP1, P4HA1, PLOD1/2, and PCOLCE* (Ito & Nagata, 2017; Lipp et al., 2023; Mai et al., 2024; Massoudi et al., 2017; Myllyharju, 2008; Sabeh et al., 2024; Vadon‐Le Goff et al., 2023). From our database, we found that genes related to collagen maturation and matrix organization, such as *LOXL2, THBS4, MMP14*, and *SERPINH1*, were significantly upregulated, whereas *P4HB* tended to be downregulated. However, some genes, such as *BMP1, P4HA1, PLOD1/2*, and *PCOLCE*, did not change (Figure 7c). These data demonstrate that resistance training enhances fibril organization and cross‐link formation, consistent with its role in strengthening and stabilizing the ECM under high mechanical stress.

In addition, the Spearman correlation showed the ECM modules connected (r ≈ 0.7–0.95, *p* < 0.001), which unified the cross‐linking enzymes (*LOX, LOXL2*), fibrillogenic regulators (SERPINH1), secretion/folding machinery (*THBS4, BMP1*), and structural collagen (*COL1A1, COL3A1, COL5A1, COL5A2, COL4A1, COL4A2*) (Figure 7d). In this network, SPARC and BGN served as hubs that bridge the fibrillar compartment and the basement membrane, showing strong correlations with *COL1A1, COL3A1*, and *COL5A2*, as well as with *COL4A1* and *COL4A2*. Remodeling proteases *MMP14* and its regulator *TIMP2* were also strongly correlated with *LOX* and *SPARC*, indicating synchronized regulation of degradation and assembly. Collectively, these trends defined the integrated transcription modules that encompassed secretion/folding (*SERPINH1*), proteolytic remodeling (*MMP14*‐*TIMP2*), and the upregulation of cross‐linking enzymes (*LOX*/*LOXL2*). Functionally, these networks bolster mechanical strength by increasing fibrillar cohesion, basement membrane integration, and mechanical stiffness, thereby facilitating hypertrophic growth and repair of microinjuries.

## 4 DISCUSSION

Most molecular studies of exercise have characterized endurance and resistance training as distinct stimuli that activate distinct biological pathways. Recent large‐scale meta‐analyses have strengthened this insight by highlighting transcription programs that tend to associate with each modality (Dickinson et al., 2018; Pillon et al., 2020). Even though some studies have reported partial overlap between two exercise modalities, the observation is predominantly based on single‐cohort analyses or pooled datasets. These methods prevent us from determining whether conserved transcriptional responses observed in previous studies genuinely exist across modalities (Jacques et al., 2025; Kelahmetoglu et al., 2020; Pietrangelo et al., 2012). The difficulty in distinguishing the universal adaptive signal from modality‐specific signals stems from differences in the protocol design, participant characteristics, and the timing of biopsy collection, which were markedly heterogeneous across human training datasets (Amar et al., 2021; Egan & Sharples, 2023; Pillon et al., 2020). Consequently, the presence and characteristics of structural adaptation signals in human skeletal muscles that are consistent across studies remain unclear.

By using a bioinformatics framework across datasets, we can observe coherent transcriptional signals that appear across the endurance and resistance cohorts we analyzed. Among the genes that consistently increase, the extracellular matrix (ECM) hub genes *COL1A1, COL1A2, COL3A1, COL4A1, COL5A1*, and *BGN* emerge as the top‐ranked hub genes across all datasets, regardless of exercise type. The recurrence of the same hub genes across modalities, albeit in different orders, indicates that the pathway centered on the ECM is not a modality‐related artifact; rather, it reflects a structural response repeatedly maintained and activated when skeletal muscle is subjected to long‐term mechanical loading. This shared signal that persists under heterogeneous conditions supports the idea that ECM remodeling represents a fundamental component of training adaptation, not just secondary or related‐context effects (Csapo et al., 2020; Gumpenberger et al., 2020; Kritikaki et al., 2021).

Beyond the shared ECM signal, both training types display the transcription feature that aligns with their physiological requirements. Endurance training explicitly increases gene expression of matrix adhesion molecules and basement membrane components, reflecting the strengthening of the pericellular architecture that supports continuous force transmission and perfusion stabilization during repeated low‐intensity loads (Kjær, 2004; Kritikaki et al., 2021). On the other hand, resistance training activates more genes involved in collagen maturation and extracellular matrix consolidation, including cross‐linking enzymes and fibrillogenesis regulators. This pattern is consistent with the requirements for matrix stabilization and microdamage repair under high mechanical loading (LeMoine et al., 2009; Scarpelli et al., 2024). This modality‐dependent specialization demonstrates that, although both training types rely on the same ECM framework, each modality recruits distinct structural modules appropriate to its dominant mechanical environment (Pillon et al., 2020).

From a biological perspective, ECM remodeling that is consistent across both modalities is logically predictable: the ECM constitutes the principal load‐bearing scaffold through which mechanical forces are transmitted, perceived, and distributed to skeletal muscle (Purslow, 2020). The strengthening of fibrillar collagen and proteoglycan is related to reinforcing the fibrillar‐basement membrane network that connects the muscle fibers, stabilizing the capillary tissues, and maintaining the niche required for satellite cell activation and tissue repair (Rayagiri et al., 2018). Because this structural continuum governs mechanotransduction and protects tendon units from strain accumulation, its adaptive remodeling is crucial for protecting the muscle from repeated loading during different training. This observation indicates that long‐term exercise requires not merely metabolic and contractile adaptations but also systematic reinforcement of the matrix architecture, which enables tissues to tolerate persistent mechanical stress (Jabre et al., 2021; Kjær, 2004; Kritikaki et al., 2021).

This finding is consistent with recent large‐scale transcriptomics studies that independently linked the ECM program with exercise adaptation. For instance, the MetaMEx meta‐analysis reported that resistance training is associated with increased gene expression related to extracellular matrix remodeling (Pillon et al., 2020). Concomitantly, the ExTraMeta identified the group of genes that respond to long‐term exercise in skeletal muscle, enriched for extracellular matrix reorganization and laminin interactions, with some collagen genes, including *COL1A1, COL4A1, COL4A2*, and *COL5A2*, forming a prominent network module (Amar et al., 2021). Beyond these meta‐analyses, several individual studies have also shown that the broad increase in various collagen isoforms and other matrix components after long‐term exercise, including aerobic protocol, selectively activates ECM biogenesis. However, previous analyses typically assessed the modality separately or relied on a combined dataset, making it unclear whether this ECM signal represents a genuine universal adaptation or a context‐dependent effect (Hjorth et al., 2015; Timmons et al., 2005). By integrating an independent cohort for endurance and resistance training and evaluating convergence across gene‐level overlap, network‐structural features, and external validation, our analysis identified ECM remodeling as a robust and reproducible transcriptional program across heterogeneous training protocols.

Even though the present study focuses on transcriptomic levels, previous research has also shown that exercise‐induced ECM remodeling can be detected at the protein level (Kritikaki et al., 2021). In endurance training, the evidence indicates increased ECM turnover rather than uniform collagen accumulation. For instance, endurance training has been reported to activate matrix metalloproteinases (MMP‐2 and MMP‐9) in human skeletal muscle, suggesting increased ECM remodeling activity (Rullman et al., 2009). In addition, studies analyzing connective tissue in relation to endurance training have shown an enhancement in collagen protein synthesis following acute training (Miller et al., 2005). However, the literature was inconsistent. One study comparing the effects of endurance training and a sedentary lifestyle showed that long‐term endurance training does not consistently increase collagen protein in muscle tissue (Mackey et al., 2005).

On the other hand, resistance training appears to cause more clearly defined ECM structural adaptation. High‐intensity resistance training has been reported to increase ECM structural protein, such as collagen XIV and tenascin‐C, in human skeletal muscle, especially around the myotendinous junction (Jakobsen et al., 2017). The proteomic study also shows that the resistance training with eccentric component enriches the protein related to the ECM‐receptor interaction pathway and structural remodeling (Du et al., 2024). Moreover, acute resistance training has been reported to increase markers of collagen remodeling and ECM turnover (Schweitzer et al., 2025). In general, these findings support the biological plausibility of the transcriptomic ECM signature identified in the present study.

The present study has some limitations. First, our analysis is based on transcriptomic data, although ECM remodeling is substantially modulated by posttranslational processes, including secretion, enzymatic modification, cross‐linking formation, and supramolecular fibril assembly, which cannot be detected at the mRNA level alone. Therefore, transcriptional alterations may not fully capture matrix mechanical properties or tissue‐level structural integrity. Second, although all datasets represent long‐term training, they were generated by different microarray platforms and collected at various times after training, which can reduce temporal resolution and blur the distinction between long‐term adaptation and transcription responses related to training sessions. The third is the cellular muscle diversity used to generate mRNA for subsequent microarray analysis across datasets. All datasets used bulk skeletal muscle biopsy that potentially contain other components, such as satellite cells, connective tissue cells, capillary endothelial cells, and perhaps other primordial cells. Therefore, the results may reflect tissue‐level remodeling involving multiple cellular components within skeletal muscle. Future studies integrating proteomic, single‐cell, and time‐resolved data will be crucial for elucidating the temporal hierarchy and mechanistic interpretation of ECM‐related adaptations.

Taken together, our findings position the extracellular matrix as a conserved structural program that underlies skeletal muscle adaptation across various training modalities. Elucidation of how this shared matrix framework integrates with modality‐specific pathways, such as adhesion‐oriented remodeling in endurance and collagen consolidation in resistance, is crucial to understanding the individual variation in transcriptomic, proteomic, and biomechanical responses that ultimately reveal the adaptive potential of human skeletal muscle.

## AUTHOR CONTRIBUTIONS

**Muhammad Isman Sandira:** Conceptualization; funding acquisition; visualization. **Firman Hasan:** Investigation; methodology. **Tsubasa Shibaguchi:** Investigation; methodology. **Hanafi Idris:** Investigation; methodology. **Mukti Mukhtar:** Investigation; methodology. **Kazumi Masuda:** Conceptualization; funding acquisition; supervision.

## FUNDING INFORMATION

This work was partially funded by JSPS KAKENHI 23K18448 and 24H00675 (to Kazumi Masuda) and 25K24318 (to Muhammad Isman Sandira).

## CONFLICT OF INTEREST STATEMENT

The authors declare no conflicts of interest.

## ETHICS STATEMENT

Not applicable.

## CONSENT

Not applicable.

## Supporting information


Data S1.

Data S2.

## Data Availability

The datasets generated and/or analyzed during the current study are available from the corresponding author upon reasonable request.

## References

[phy270874-bib-0001] Ahmad, K. , Shaikh, S. , Ahmad, S. S. , Lee, E. J. , & Choi, I. (2020). Cross‐talk between extracellular matrix and skeletal muscle: Implications for myopathies. Frontiers in Pharmacology, 11, 142. 32184725 10.3389/fphar.2020.00142 PMC7058629

[phy270874-bib-0002] Amar, D. , Lindholm, M. E. , Norrbom, J. , Wheeler, M. T. , Rivas, M. A. , & Ashley, E. A. (2021). Time trajectories in the transcriptomic response to exercise ‐ a meta‐analysis. Nature Communications, 12(1), 3471. 10.1038/s41467-021-23579-x PMC8190306 34108459

[phy270874-bib-0003] Bader, G. D. , & Hogue, C. W. V. (2003). An automated method for finding molecular complexes in large protein interaction networks. BMC Bioinformatics, 4, 2. 12525261 10.1186/1471-2105-4-2 PMC149346

[phy270874-bib-0004] Befroy, D. E. , Petersen, K. F. , Dufour, S. , Mason, G. F. , Rothman, D. L. , & Shulman, G. I. (2008). Increased substrate oxidation and mitochondrial uncoupling in skeletal muscle of endurance‐trained individuals. Proceedings of the National Academy of Sciences of the United States of America, 105(43), 16701–16706. 18936488 10.1073/pnas.0808889105 PMC2570428

[phy270874-bib-0005] Beiter, T. , Zügel, M. , Hudemann, J. , Schild, M. , Fragasso, A. , Burgstahler, C. , Krüger, K. , Mooren, F. C. , Steinacker, J. M. , & Nieß, A. M. (2024). The acute, short‐, and long‐term effects of endurance exercise on skeletal muscle transcriptome profiles. International Journal of Molecular Sciences, 25(5), 2881. 38474128 10.3390/ijms25052881 PMC10932090

[phy270874-bib-0006] Chin, C. H. , Chen, S. H. , Wu, H. H. , Ho, C. W. , Ko, M. T. , & Lin, C. Y. (2014). cytoHubba: Identifying hub objects and sub‐networks from complex interactome. BMC Systems Biology, 8(Suppl 4), S11. 25521941 10.1186/1752-0509-8-S4-S11 PMC4290687

[phy270874-bib-0007] Cho, H. J. , Lee, Y. S. , Kim, D. A. , Moon, S. A. , Lee, S. E. , Lee, S. H. , & Koh, J. M. (2022). Lumican, an exerkine, protects against skeletal muscle loss. International Journal of Molecular Sciences, 23(17), 10031. 36077426 10.3390/ijms231710031 PMC9456076

[phy270874-bib-0008] Csapo, R. , Gumpenberger, M. , & Wessner, B. (2020). Skeletal muscle extracellular matrix—what do we know about its composition, regulation, and physiological roles? A narrative review. Frontiers in Physiology, 11, 253. 32265741 10.3389/fphys.2020.00253 PMC7096581

[phy270874-bib-0009] Damas, F. , Phillips, S. M. , Libardi, C. A. , Vechin, F. C. , Lixandrão, M. E. , Jannig, P. R. , Costa, L. A. R. , Bacurau, A. V. , Snijders, T. , Parise, G. , Tricoli, V. , Roschel, H. , & Ugrinowitsch, C. (2016). Resistance training‐induced changes in integrated myofibrillar protein synthesis are related to hypertrophy only after attenuation of muscle damage. The Journal of Physiology, 594(18), 5209–5222. 27219125 10.1113/JP272472 PMC5023708

[phy270874-bib-0010] Damas, F. , Ugrinowitsch, C. , Libardi, C. A. , Jannig, P. R. , Hector, A. J. , McGlory, C. , Lixandrão, M. E. , Vechin, F. C. , Montenegro, H. , Tricoli, V. , Roschel, H. , & Phillips, S. M. (2018). Resistance training in young men induces muscle transcriptome‐wide changes associated with muscle structure and metabolism refining the response to exercise‐induced stress. European Journal of Applied Physiology, 118(12), 2607–2616. 30196447 10.1007/s00421-018-3984-y

[phy270874-bib-0011] Dickinson, J. M. , D'Lugos, A. C. , Naymik, M. A. , Siniard, A. L. , Wolfe, A. J. , Curtis, D. P. , Huentelman, M. J. , & Carroll, C. C. (2018). Transcriptome response of human skeletal muscle to divergent exercise stimuli. Journal of Applied Physiology (Bethesda, MD: 1985), 124(6), 1529–1540. 29543133 10.1152/japplphysiol.00014.2018

[phy270874-bib-0012] Doncheva, N. T. , Morris, J. H. , Gorodkin, J. , & Jensen, L. J. (2019). Cytoscape stringApp: Network analysis and visualization of proteomics data. Journal of Proteome Research, 18(2), 623–632. 30450911 10.1021/acs.jproteome.8b00702 PMC6800166

[phy270874-bib-0013] Du, J. , Yun, H. , Wang, H. , Bai, X. , Su, Y. , Ge, X. , Wang, Y. , Gu, B. , Zhao, L. , Yu, J. G. , & Song, Y. (2024). Proteomic profiling of muscular adaptations to short‐term concentric versus eccentric exercise training in humans. Molecular and Cellular Proteomics, 23(4), 100748. 38493954 10.1016/j.mcpro.2024.100748 PMC11017286

[phy270874-bib-0014] Egan, B. , & Sharples, A. P. (2023). Molecular responses to acute exercise and their relevance for adaptations in skeletal muscle to exercise training. Physiological Reviews, 103(3), 2057–2170. 36395350 10.1152/physrev.00054.2021

[phy270874-bib-0015] Gehlert, S. , Weinisch, P. , Römisch‐Margl, W. , Jaspers, R. T. , Artati, A. , Adamski, J. , Dyar, K. A. , Aussieker, T. , Jacko, D. , Bloch, W. , Wackerhage, H. , & Kastenmüller, G. (2022). Effects of acute and chronic resistance exercise on the skeletal muscle metabolome. Metabolites, 12(5), 445. 35629949 10.3390/metabo12050445 PMC9142957

[phy270874-bib-0016] Gumpenberger, M. , Wessner, B. , Graf, A. , Narici, M. V. , Fink, C. , Braun, S. , Hoser, C. , Blazevich, A. J. , & Csapo, R. (2020). Remodeling the skeletal muscle extracellular matrix in older age—Effects of acute exercise stimuli on gene expression. International Journal of Molecular Sciences, 21(19), 7089. 32992998 10.3390/ijms21197089 PMC7583913

[phy270874-bib-0017] Hashmi, S. , & Marinkovich, M. P. (2011). Molecular organization of the basement membrane zone. Clinics in Dermatology, 29(4), 398–411. 21679867 10.1016/j.clindermatol.2011.01.009

[phy270874-bib-0018] Hjorth, M. , Norheim, F. , Meen, A. J. , Pourteymour, S. , Lee, S. , Holen, T. , Jensen, J. , Birkeland, K. I. , Martinov, V. N. , Langleite, T. M. , Eckardt, K. , Drevon, C. A. , & Kolset, S. O. (2015). The effect of acute and long‐term physical activity on extracellular matrix and serglycin in human skeletal muscle. Physiological Reports, 3(8), e12473. 26290530 10.14814/phy2.12473 PMC4562559

[phy270874-bib-0019] Hohenester, E. , Sasaki, T. , Giudici, C. , Farndale, R. W. , & Bächinger, H. P. (2008). Structural basis of sequence‐specific collagen recognition by SPARC. Proceedings of the National Academy of Sciences of the United States of America, 105(47), 18273–18277. 19011090 10.1073/pnas.0808452105 PMC2587565

[phy270874-bib-0020] Hoppeler, H. , Baum, O. , Lurman, G. , & Mueller, M. (2011). Molecular mechanisms of muscle plasticity with exercise. Comprehensive Physiology, 1(3), 1383–1412. 23733647 10.1002/cphy.c100042

[phy270874-bib-0021] Huang, D. W. , Sherman, B. T. , & Lempicki, R. A. (2009a). Systematic and integrative analysis of large gene lists using DAVID bioinformatics resources. Nature Protocols, 4(1), 44–57. 19131956 10.1038/nprot.2008.211

[phy270874-bib-0022] Huang, D. W. , Sherman, B. T. , & Lempicki, R. A. (2009b). Bioinformatics enrichment tools: Paths toward the comprehensive functional analysis of large gene lists. Nucleic Acids Research, 37(1), 1–13. 19033363 10.1093/nar/gkn923 PMC2615629

[phy270874-bib-0023] Ida, T. , Kaku, M. , Kitami, M. , Terajima, M. , Rocabado, J. M. R. , Akiba, Y. , Rosales Rocabado, J. M. , Nagasawa, M. , Yamauchi, M. , & Uoshima, K. (2018). Extracellular matrix with defective collagen cross‐linking affects the differentiation of bone cells. PLoS One, 13(9), e0204306. 30252876 10.1371/journal.pone.0204306 PMC6155528

[phy270874-bib-0024] Ito, S. , & Nagata, K. (2017). Biology of Hsp47 (serpin H1), a collagen‐specific molecular chaperone. Seminars in Cell & Developmental Biology, 62, 142–151. 27838364 10.1016/j.semcdb.2016.11.005

[phy270874-bib-0025] Jabre, S. , Hleihel, W. , & Coirault, C. (2021). Nuclear mechanotransduction in skeletal muscle. Cells, 10(2), 318. 33557157 10.3390/cells10020318 PMC7913907

[phy270874-bib-0026] Jacques, M. , Landen, S. , Sharples, A. P. , Garnham, A. , Schittenhelm, R. , Steele, J. , Heikkinen, A. , Sillanpää, E. , Ollikainen, M. , Broatch, J. , Zarekookandeh, N. , Hanson, O. , Ekström, O. , Asplund, O. , Lamon, S. , Alexander, S. E. , Smith, C. , Bauer, C. , Woessner, M. N. , … Eynon, N. (2025). Molecular landscape of sex‐ and modality‐specific exercise adaptation in human skeletal muscle through large‐scale multi‐omics integration. Cell Reports, 44(6), 115750. 40445834 10.1016/j.celrep.2025.115750

[phy270874-bib-0027] Jakobsen, J. R. , Mackey, A. L. , Knudsen, A. B. , Koch, M. , Kjær, M. , & Krogsgaard, M. R. (2017). Composition and adaptation of human myotendinous junction and neighboring muscle fibers to heavy resistance training. Scandinavian Journal of Medicine & Science in Sports, 27(12), 1547–1559. 27781307 10.1111/sms.12794

[phy270874-bib-0028] Kelahmetoglu, Y. , Jannig, P. R. , Cervenka, I. , Koch, L. G. , Britton, S. L. , Zhou, J. , Wang, H. , Robinson, M. M. , Nair, K. S. , & Ruas, J. L. (2020). Comparative analysis of skeletal muscle transcriptional signatures associated with aerobic exercise capacity or response to training in humans and rats. Frontiers in Endocrinology (Lausanne), 11, 591476. 10.3389/fendo.2020.591476 PMC7649134 33193103

[phy270874-bib-0029] Keller, P. , Vollaard, N. B. , Gustafsson, T. , Gallagher, I. J. , Sundberg, C. J. , Rankinen, T. , Britton, S. L. , Bouchard, C. , Koch, L. G. , & Timmons, J. A. (2011). A transcriptional map of the impact of endurance exercise training on skeletal muscle phenotype. Journal of Applied Physiology (Bethesda, MD: 1985), 110(1), 46–59. 20930125 10.1152/japplphysiol.00634.2010 PMC3253010

[phy270874-bib-0030] Kjær, M. (2004). Role of extracellular matrix in adaptation of tendon and skeletal muscle to mechanical loading. Physiological Reviews, 84(2), 649–698. 15044685 10.1152/physrev.00031.2003

[phy270874-bib-0031] Koma, R. , Shibaguchi, T. , Yamada, T. , Nonaka, Y. , Yamazaki, A. , Jue, T. , & Masuda, K. (2024). Endurance training increases mitochondrial myoglobin and enhances its interaction with complex IV in rat plantaris muscle. Acta Physiologica (Oxford), 240(5), e14139. 10.1111/apha.14139 38509816

[phy270874-bib-0032] Kritikaki, E. , Asterling, R. , Ward, L. , Padget, K. , Barreiro, E. , & Simoes, D. C. M. (2021). Exercise training‐induced extracellular matrix protein adaptation in locomotor muscles: A systematic review. Cells, 10(5), 1022. 33926070 10.3390/cells10051022 PMC8146973

[phy270874-bib-0033] Lavin, K. M. , Bell, M. B. , JS, M. A. , Peck, B. D. , Walton, R. G. , Windham, S. T. , Tuggle, S. C. , Long, D. E. , Kern, P. A. , Peterson, C. A. , & Bamman, M. M. (2021). Muscle transcriptional networks linked to resistance exercise training hypertrophic response heterogeneity. Physiological Genomics, 53(5), 206–221. 33870722 10.1152/physiolgenomics.00154.2020 PMC8424535

[phy270874-bib-0034] LeMoine, J. K. , Lee, J. D. , & Trappe, T. A. (2009). Impact of sex and chronic resistance training on human patellar tendon dry mass, collagen content, and collagen cross‐linking. American Journal of Physiology. Regulatory, Integrative and Comparative Physiology, 296(1), R119–R124. 18945950 10.1152/ajpregu.90607.2008 PMC2636977

[phy270874-bib-0035] Lindholm, M. E. , Giacomello, S. , Werne Solnestam, B. , Fischer, H. , Huss, M. , Kjellqvist, S. , & Sundberg, C. J. (2016). The impact of endurance training on human skeletal muscle memory, global isoform expression and novel transcripts. PLoS Genetics, 12(9), e1006294. 27657503 10.1371/journal.pgen.1006294 PMC5033478

[phy270874-bib-0036] Lipp, S. N. , Jacobson, K. R. , Colling, H. A. , Tuttle, T. G. , Miles, D. T. , KP, M. C. , & Calve, S. (2023). Mechanical loading is required for initiation of extracellular matrix deposition at the developing murine myotendinous junction. Matrix Biology, 116, 28–48. 36709857 10.1016/j.matbio.2023.01.003 PMC10218368

[phy270874-bib-0037] Liu, D. , Sartor, M. A. , Nader, G. A. , Gutmann, L. , Treutelaar, M. K. , Pistilli, E. E. , IglayReger, H. B. , Burant, C. F. , Hoffman, E. P. , & Gordon, P. M. (2010). Skeletal muscle gene expression in response to resistance exercise: Sex specific regulation. BMC Genomics, 11, 659. 21106073 10.1186/1471-2164-11-659 PMC3091777

[phy270874-bib-0038] Mackey, A. L. , Donnelly, A. E. , & Roper, H. P. (2005). Muscle connective tissue content of endurance‐trained and inactive individuals. Scandinavian Journal of Medicine & Science in Sports, 15(6), 402–408. 16293152 10.1111/j.1600-0838.2005.00449.x

[phy270874-bib-0039] Mai, Z. , Lin, Y. , Lin, P. , Zhao, X. , & Cui, L. (2024). Modulating extracellular matrix stiffness: A strategic approach to boost cancer immunotherapy. Cell Death & Disease, 15(5), 372. 38693104 10.1038/s41419-024-06697-4 PMC11063215

[phy270874-bib-0040] Massoudi, D. , Germer, C. J. , Glisch, J. M. , & Greenspan, D. S. (2017). Procollagen C‐proteinase enhancer 1 (PCPE‐1) functions as an anti‐angiogenic factor and enhances epithelial recovery in injured cornea. Cell and Tissue Research, 370(3), 461–476. 28936615 10.1007/s00441-017-2689-6 PMC5705274

[phy270874-bib-0041] McGlory, C. , Devries, M. C. , & Phillips, S. M. (2017). Skeletal muscle and resistance exercise training: The role of protein synthesis in recovery and remodeling. Journal of Applied Physiology (Bethesda, MD: 1985), 122(3), 541–548. 27742803 10.1152/japplphysiol.00613.2016 PMC5401959

[phy270874-bib-0042] Meinen, S. , Barzaghi, P. , Lin, S. , Lochmüller, H. , & Ruegg, M. A. (2007). Linker molecules between laminins and dystroglycan ameliorate laminin‐α2‐deficient muscular dystrophy at all disease stages. The Journal of Cell Biology, 176(7), 979–993. 17389231 10.1083/jcb.200611152 PMC2064083

[phy270874-bib-0043] Miller, B. F. , Olesen, J. L. , Hansen, M. , Døssing, S. , Crameri, R. M. , Welling, R. J. , Langberg, H. , Flyvbjerg, A. , Kjaer, M. , Babraj, J. A. , Smith, K. , & Rennie, M. J. (2005). Coordinated collagen and muscle protein synthesis in human patella tendon and quadriceps muscle after exercise. The Journal of Physiology, 567(3), 1021–1033. 16002437 10.1113/jphysiol.2005.093690 PMC1474228

[phy270874-bib-0044] Murach, K. A. , & Bagley, J. R. (2025). A primer on global molecular responses to exercise in skeletal muscle: Omics in focus. Journal of Sport and Health Science, 15, S2095‐2546(25)00007–9. 10.1016/j.jshs.2025.101029 PMC12816910 39961420

[phy270874-bib-0045] Myllyharju, J. (2008). Prolyl 4‐hydroxylases, key enzymes in the synthesis of collagens and regulation of the response to hypoxia, and their roles as treatment targets. Annals of Medicine, 40(6), 402–417. 19160570 10.1080/07853890801986594

[phy270874-bib-0046] Pathan, M. , Keerthikumar, S. , Ang, C. S. , Gangoda, L. , Quek, C. Y. J. , Williamson, N. A. , Mouradov, D. , Sieber, O. M. , Simpson, R. J. , Salim, A. , Bacic, A. , Hill, A. F. , Stroud, D. A. , Ryan, M. T. , Agbinya, J. I. , Mariadason, J. M. , Burgess, A. W. , & Mathivanan, S. (2015). FunRich: An open access standalone functional enrichment and interaction network analysis tool. Proteomics, 15(15), 2597–2601. 25921073 10.1002/pmic.201400515

[phy270874-bib-0047] Pietrangelo, T. , Mancinelli, R. , Doria, C. , Di Tano, G. , Loffredo, B. , Fanò‐Illic, G. , & Fulle, S. (2012). Endurance and resistance training modifies the transcriptional profile of the vastus lateralis skeletal muscle in healthy elderly subjects. Sport Sciences for Health, 7(1), 19–27.

[phy270874-bib-0048] Pillon, N. J. , Gabriel, B. M. , Dollet, L. , Smith, J. A. B. , Sardón Puig, L. , Botella, J. , Bishop, D. J. , Krook, A. , & Zierath, J. R. (2020). Transcriptomic profiling of skeletal muscle adaptations to exercise and inactivity. Nature Communications, 11(1), 470. 10.1038/s41467-019-13869-w PMC6981202 31980607

[phy270874-bib-0049] Popov, D. V. , Makhnovskii, P. A. , Shagimardanova, E. I. , Gazizova, G. R. , Lysenko, E. A. , Gusev, O. A. , & Vinogradova, O. L. (2019). Contractile activity‐specific transcriptome response to acute endurance exercise and training in human skeletal muscle. American Journal of Physiology. Endocrinology and Metabolism, 316(4), E605–E614. 30779632 10.1152/ajpendo.00449.2018

[phy270874-bib-0050] Purslow, P. P. (2020). The structure and role of intramuscular connective tissue in muscle function. Frontiers in Physiology, 11, 495. 32508678 10.3389/fphys.2020.00495 PMC7248366

[phy270874-bib-0051] Rayagiri, S. S. , Ranaldi, D. , Raven, A. , Mohamad Azhar, N. I. F. , Lefebvre, O. , Zammit, P. S. , & Borycki, A. G. (2018). Basal lamina remodeling at the skeletal muscle stem cell niche mediates stem cell self‐renewal. Nature Communications, 9(1), 1075. 10.1038/s41467-018-03425-3 PMC5852002 29540680

[phy270874-bib-0052] Ritchie, M. E. , Phipson, B. , Wu, D. , Hu, Y. , Law, C. W. , Shi, W. , & Smyth, G. K. (2015). Limma powers differential expression analyses for RNA‐sequencing and microarray studies. Nucleic Acids Research, 43(7), e47. 25605792 10.1093/nar/gkv007 PMC4402510

[phy270874-bib-0053] Robinson, M. M. , Dasari, S. , Konopka, A. R. , Johnson, M. L. , Manjunatha, S. , Esponda, R. R. , Carter, R. E. , Lanza, I. R. , & Nair, K. S. (2017). Enhanced protein translation underlies improved metabolic and physical adaptations to different exercise training modes in young and old humans. Cell Metabolism, 25(3), 581–592. 28273480 10.1016/j.cmet.2017.02.009 PMC5423095

[phy270874-bib-0054] Roman, W. , Martins, J. P. , & Gomes, E. R. (2018). Local arrangement of fibronectin by myofibroblasts governs peripheral nuclear positioning in muscle cells. Developmental Cell, 46(1), 102–111.e6. 29937388 10.1016/j.devcel.2018.05.031 PMC6035285

[phy270874-bib-0055] Rønning, S. B. , Carlson, C. R. , Stang, E. , Kolset, S. O. , Hollung, K. , & Pedersen, M. E. (2015). Syndecan‐4 regulates muscle differentiation and is internalized from the plasma membrane during myogenesis. PLoS One, 10(6), e0129288. 26068620 10.1371/journal.pone.0129288 PMC4467083

[phy270874-bib-0056] Rullman, E. , Norrbom, J. , Strömberg, A. , Wågsäter, D. , Rundqvist, H. , Haas, T. , & Gustafsson, T. (2009). Endurance exercise activates matrix metalloproteinases in human skeletal muscle. Journal of Applied Physiology (1985), 106(3), 804–812. 10.1152/japplphysiol.90872.2008 19131480

[phy270874-bib-0057] Sabeh, F. , Li, X. Y. , Olson, A. W. , Botvinick, E. , Kurup, A. , Gimenez, L. E. , Cho, J. S. , & Weiss, S. J. (2024). Mmp14‐dependent remodeling of the pericellular‐dermal collagen interface governs fibroblast survival. The Journal of Cell Biology, 223(9), e202312091. 38990714 10.1083/jcb.202312091 PMC11244150

[phy270874-bib-0058] Scarpelli, M. C. , Bergamasco, J. G. A. , Godwin, J. S. , Mesquita, P. H. C. , Chaves, T. S. , Silva, D. G. , Bittencourt, D. , Dias, N. F. , Medalha Junior, R. A. , Carello Filho, P. C. , Angleri, V. , Costa, L. A. R. , Kavazis, A. N. , Ugrinowitsch, C. , Roberts, M. D. , & Libardi, C. A. (2024). Resistance training‐induced changes in muscle proteolysis and extracellular matrix remodeling biomarkers in the untrained and trained states. European Journal of Applied Physiology, 124(9), 2749–2762. 38653795 10.1007/s00421-024-05484-5

[phy270874-bib-0059] Schweitzer, A. M. , Koehle, M. S. , Fliss, M. D. , & Mitchell, C. J. (2025). Collagen remodeling increases after acute resistance exercise in healthy skeletal muscle irrespective of age. American Journal of Physiology. Cell Physiology, 329(1), C68–C81. 40454703 10.1152/ajpcell.00992.2024

[phy270874-bib-0060] Steffensen, L. B. , Stubbe, J. , Lindholt, J. S. , Beck, H. C. , Overgaard, M. , Bloksgaard, M. , Genovese, F. , Holm Nielsen, S. , Tha, M. L. T. , Bang‐Moeller, S. K. , Hong Lin, M. K. T. , Larsen, J. H. , Hansen, D. R. , Jones, G. T. , Bown, M. J. , Karsdal, M. A. , & Rasmussen, L. M. (2021). Basement membrane collagen IV deficiency promotes abdominal aortic aneurysm formation. Scientific Reports, 11(1), 12903. 34145342 10.1038/s41598-021-92303-y PMC8213747

[phy270874-bib-0061] Stokes, T. , Timmons, J. A. , Crossland, H. , Tripp, T. R. , Murphy, K. , McGlory, C. , Mitchell, C. J. , Oikawa, S. Y. , Morton, R. W. , Phillips, B. E. , Baker, S. K. , Atherton, P. J. , Wahlestedt, C. , & Phillips, S. M. (2020). Molecular transducers of human skeletal muscle remodeling under different loading states. Cell Reports, 32(5), 107980. 32755574 10.1016/j.celrep.2020.107980 PMC7408494

[phy270874-bib-0062] Sun, L. , Luan, J. , Wang, J. , Li, X. , Zhang, W. , Ji, X. , Liu, L. , Wang, R. , & Xu, B. (2025). GEPREP: A comprehensive data atlas of RNA‐seq‐based gene expression profiles of exercise responses. Journal of Sport and Health Science, 14(1), 100992. 39341494 10.1016/j.jshs.2024.100992 PMC11863345

[phy270874-bib-0063] Szklarczyk, D. , Kirsch, R. , Koutrouli, M. , Nastou, K. , Mehryary, F. , Hachilif, R. , Gable, A. L. , Fang, T. , Doncheva, N. T. , Pyysalo, S. , Bork, P. , Jensen, L. J. , & von Mering, C. (2023). The STRING database in 2023: Protein‐protein association networks and functional enrichment analyses for any sequenced genome of interest. Nucleic Acids Research, 51(D1), D638–D646. 36370105 10.1093/nar/gkac1000 PMC9825434

[phy270874-bib-0064] Tang, D. , Chen, M. , Huang, X. , Zhang, G. , Zeng, L. , Zhang, G. , Wu, S. , & Wang, Y. (2023). SRplot: A free online platform for data visualization and graphing. PLoS One, 18(11), e0294236. 37943830 10.1371/journal.pone.0294236 PMC10635526

[phy270874-bib-0065] Thorsteinsdóttir, S. , Deries, M. , Cachaço, A. S. , & Bajanca, F. (2011). The extracellular matrix dimension of skeletal muscle development. Developmental Biology, 354(2), 191–207. 21420400 10.1016/j.ydbio.2011.03.015

[phy270874-bib-0066] Timmons, J. A. , Jansson, E. , Fischer, H. , Gustafsson, T. , Greenhaff, P. L. , Ridden, J. , Rachman, J. , & Sundberg, C. J. (2005). Modulation of extracellular matrix genes reflects the magnitude of physiological adaptation to aerobic exercise training in humans. BMC Biology, 3, 19. 16138928 10.1186/1741-7007-3-19 PMC1224855

[phy270874-bib-0067] Turan, N. , Kalko, S. , Stincone, A. , Clarke, K. , Sabah, A. , Howlett, K. , Curnow, S. J. , Rodriguez, D. A. , Cascante, M. , O'Neill, L. , Egginton, S. , Roca, J. , & Falciani, F. (2011). A systems biology approach identifies molecular networks defining skeletal muscle abnormalities in chronic obstructive pulmonary disease. PLoS Computational Biology, 7(9), e1002129. 21909251 10.1371/journal.pcbi.1002129 PMC3164707

[phy270874-bib-0068] Urciuolo, A. , Quarta, M. , Morbidoni, V. , Gattazzo, F. , Molon, S. , Grumati, P. , Montemurro, F. , Tedesco, F. S. , Blaauw, B. , Cossu, G. , Vozzi, G. , Rando, T. A. , & Bonaldo, P. (2013). Collagen VI regulates satellite cell self‐renewal and muscle regeneration. Nature Communications, 4, 1964. 10.1038/ncomms2964 PMC3682802 23743995

[phy270874-bib-0069] Vadon‐Le Goff, S. , Tessier, A. , Napoli, M. , Dieryckx, C. , Bauer, J. , Dussoyer, M. , Lagoutte, P. , Peyronnel, C. , Essayan, L. , Kleiser, S. , & Tueni, N. (2023). Identification of PCPE‐2 as the endogenous specific inhibitor of human BMP‐1/tolloid‐like proteinases. Nature Communications, 14(1), 8020. 10.1038/s41467-023-43401-0 PMC10696041 38049428

[phy270874-bib-0070] Vann, C. G. , Roberson, P. A. , Osburn, S. C. , Mumford, P. W. , Romero, M. A. , Fox, C. D. , Moore, J. H. , Haun, C. , Beck, D. T. , Moon, J. R. , Kavazis, A. N. , Young, K. C. , Badisa, V. L. D. , Mwashote, B. M. , Ibeanusi, V. , Singh, R. K. , & Roberts, M. D. (2020). Skeletal muscle myofibrillar protein abundance is higher in resistance‐trained men, and aging in the absence of training may have an opposite effect. Sports (Basel), 8(1), 7. 31936810 10.3390/sports8010007 PMC7022975

[phy270874-bib-0071] Viggars, M. R. , Sutherland, H. , Lanmüller, H. , Schmoll, M. , Bijak, M. , & Jarvis, J. C. (2023). Adaptation of the transcriptional response to resistance exercise over 4 weeks of daily training. FASEB Journal, 37(1), e22686. 36468768 10.1096/fj.202201418R PMC13281843

[phy270874-bib-0072] Wenstrup, R. J. , Florer, J. B. , Brunskill, E. W. , Bell, S. M. , Chervoneva, I. , & Birk, D. E. (2004). Type V collagen controls the initiation of collagen fibril assembly. The Journal of Biological Chemistry, 279(51), 53331–53337. 15383546 10.1074/jbc.M409622200

